# Experimental stone clearance with in‐scope suction and flexible and navigable suction access sheaths

**DOI:** 10.1111/bju.16849

**Published:** 2025-07-29

**Authors:** Richard Menzies‐Wilson, Thijs Ruiken, Benjamin Turney

**Affiliations:** ^1^ Nuffield Department of Surgery University of Oxford Oxford UK

**Keywords:** suction, direct in‐scope suction, DISS, access sheath, FANS, ureteroscopy

## Abstract

**Objective:**

To perform benchtop experiments using direct in‐scope suction (DISS) ureteroscopes (Pusen Medical, Zhuhai, China; 3.6‐Ch and 5.1‐Ch working channels) and flexible and navigable suction (FANS) ureteric access sheaths (ClearPetra; Wellead, Guangzhou, China) to establish their ability to clear stones of different diameters.

**Methods:**

For each experimental group, we conducted a series of experiments using mixes of progressively larger stone particles (<63, 63–125, 125–250, 250–500, 500–1000 and 1000–2000 μm) until suction through the respective lumen failed to achieve complete stone clearance; the first experiment's stone mix contained particles <63 μm; the second experiment's stone mix contained particles <63 μm + 63–125 μm; the third experiment's stone mix contained particles <63 μm + 63–125 μm + 125–250 μm, and so on, up to 2000 μm. The stone mixes were introduced in aliquots over the course of 30 min. In the first experimental group, either a 3.6‐Ch or a 5.1‐Ch working channel DISS ureteroscope (with a 200‐μm laser) was used to aspirate stones. In the second experimental group, either an empty 5.1‐Ch working channel DISS ureteroscope or an 11/13‐Ch FANS with a 9.5‐Ch ureteroscope (intermittently withdrawn) was used to aspirate stones.

**Results:**

The use of DISS through a 3.6‐Ch working channel (with laser fibre) cleared stones up to 250 μm. DISS through a 5.1‐Ch working channel cleared stones up to 500 μm with and without an indwelling laser fibre. FANS cleared all stone sizes tested (up to 2000 μm).

**Conclusions:**

During laser lithotripsy, DISS can aspirate ‘dust’ particles (<250 μm), which are known to most affect vision. However, particles >250 μm and >500 μm (with 3.6‐Ch and 5.1‐Ch working channels) may block the ureteroscope. The use of FANS, through successful clearance of larger fragments, may facilitate clearance of larger fragments.

**Abbreviations**
DISS: direct in‐scope suction
FANS: flexible and navigable suction
SFR: stone‐free rate

## Introduction

Ureteroscopy has evolved significantly over the years, with advancements in technology improving its efficacy and safety. Among these innovations, the application of suction may be a significant development, offering the ability to aspirate stone fragments to improve stone‐free rates (SFRs) [1].

Traditionally, either dusting or fragmenting have been advocated for treating kidney stones [2]. In the dusting method, the laser is used to break the stone into small enough particles to pass spontaneously in the urine [2], while fragmenting involves removing larger fragments individually using a basket via the ureteroscope to capture and withdraw stone pieces [2]. Complete stone clearance (zero fragments) is frequently not achieved with either method; SFRs are reported to be 74%–97% [3]. Increasingly, there is an understanding that leaving behind any residual fragments poses a potential nidus for stone regrowth and increases retreatment rates [4, 5, 6].

Recently, in an attempt to increase SFRs, suction devices have been introduced [7]. Suction can be applied either through a flexible and navigable suction (FANS) ureteric access sheath or by direct in‐scope suction (DISS), whereby stones are aspirated through the ureteroscope working channel [7].

The FANS ureteric access sheath differs from a normal access sheath in two distinct ways [8]. Firstly, it has an additional port allowing suction to be applied to its working channel [8]. Secondly, it has a flexible tip allowing it to be manoeuvred past the PUJ and into the renal pelvis or renal calyces [8].

With DISS, fluid and stones are aspirated directly through the working channel (i.e. the irrigation channel) of the ureteroscope. The working channel of most current commercially available suction flexible ureteroscopes is 3.6 Ch. Some authors have suggested that particles up to 250 μm could be aspirated through a 3.6‐Ch working channel [9]. Theoretically, increasing the working channel cross‐sectional surface area may increase the size of the stone size that can be aspirated, and recently, a DISS ureteroscope with a 5.1‐Ch working channel has become commercially available [10].

In a series of benchtop experiments we aimed:

1. to establish the maximum stone particle sizes that could be cleared using DISS through a 3.6‐Ch or 5.1‐Ch working channel with an indwelling 200‐μm laser fibre; and
2. to establish the maximum stone particle sizes that could be cleared using DISS through an empty 5.1‐Ch working channel vs suction through an 11/13‐Ch FANS ureteric access sheath.

## Methods

We calculated the cross‐sectional area of a 3.6‐Ch and 5.1‐Ch working channel, with and without a 200‐μm laser fibre (SOLTIVE™; Olympus Corporation) in the working channel. Notably, the 200‐μm laser fibre has an outer core width of 400 μm [11]. We also calculated the cross‐sectional area of an 11/13‐Ch FANS ureteric access sheath.

A quartz model has been developed by our laboratory, as a surrogate for renal stones, to investigate the effect of ureteroscopy flow on stone particle clearance [12]. Quartz stones have a similar density (2.6 g/cm^3^ vs 1.7–2 g/cm^3^), shape and insolubility to those of calcium oxalate monohydrate stones and therefore represent a reasonable surrogate [12]. The quartz stones were sieved into categories of different diameters using stacked test sieves (Endecott Ltd, UK): <63, 63–125, 125–250, 250–500, 500–1000 and 1000–2000 μm.

Stone samples were created to simulate the range of particle sizes produced by ‘dusting’ a 1‐cm calcium oxalate monohydrate stone with a Lumenis Pulse 120H holmium laser system with MOSES Technology, based on our laboratory's previous research [12]. The stone diameters (and corresponding masses) were as follows: <63 μm (0.55 g), 63–125 μm (0.15 g), 125–250 μm (0.05 g), 250–500 μm (0.05 g), 500–1000 μm (0.1 g), and 1000–2000 μm (0.1 g) [12]. For each of the experimental groups, we performed a series of experiments using mixes of progressively larger stone particles until a mixture failed to be cleared. For instance, the initial stone mix contained only particles <63 μm (0.55 g); if cleared, the next mix contained particles <63 μm (0.55 g) + particles 63–125 μm (0.15 g); if cleared, the next mix contained particles <63 μm (0.55 g) + particles 63–125 μm (0.15 g) + particles 125–250 μm (0.05 g), and so on until a mixture failed to be cleared (Table 1). The results detailed which stone mixes could and could not be fully cleared.

**Table 1:** Masses of different‐sized particles used in the stone mixes of sequentially increasing sizes.

Particle sizes	Stone mix up to 63 μm	Stone mix up to 125 μm	Stone mix up to 250 μm	Stone mix up to 500 μm	Stone mix up to 1000 μm	Stone mix up to 2000 μm
<63 μm	0.55 g	0.55 g	0.55 g	0.55 g	0.55 g	0.55 g
63–125 μm		0.15 g	0.15 g	0.15 g	0.15 g	0.15 g
125–250 μm			0.05 g	0.05 g	0.05 g	0.05 g
250–500 μm				0.05 g	0.05 g	0.05 g
500–1000 μm					0.1 g	0.1 g
1000–2000 μm						0.1 g

**Fig. 1:**
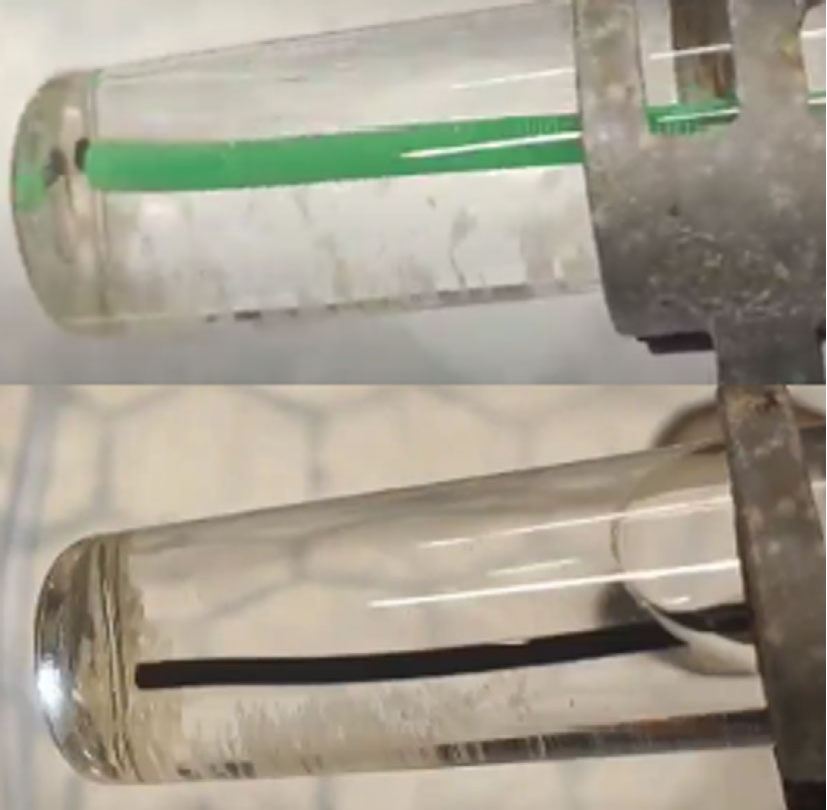
Experimental set‐up: aspiration of stone particles from a glass vial at 30° incline with a flexible and navigable suction access sheath (top) and in‐scope suction (bottom).

In the first experiment we investigated the maximum stone diameter that could be aspirated through a 3.6‐Ch and 5.1‐Ch working channel containing a laser fibre. Aspiration through the ureteroscopes with a laser fibre *in situ* mimics the clinical setting when the surgeon is lasering and aspirating intra‐operatively. A 7.5‐Ch DISS ureteroscope (PU3033AH; Pusen Medical, Zhuhai, China) with a 3.6‐Ch working channel or a prototype 9.2‐Ch DISS ureteroscope with a 5.1‐Ch working channel (Pusen Medical) was placed within a glass vial at an incline of 30**°** (Fig. 1). The 200‐μm laser fibre (Olympus Corporation) was placed in the working channel, protruding 2 mm beyond the end of the scope. Irrigation pressure was set at 200 mmHg with an irrigation pressure bag. Suction was set at 400 mmHg (to match Pusen's prototype suction pump) with a medical vacuum suction machine (SAM12; MG Electric Ltd, Colchester, UK) [13]. Throughout the experiment, irrigation and suction were alternated to fill and empty the vial to 40 mL (to replicate the intermittent filling and emptying of the renal collecting system). Importantly, the irrigation inflow tubing was clamped when suctioning through the scope to prevent suction of irrigation fluid from the irrigation bag.

The stone mix was introduced gradually into the vial, to replicate the gradual lasering of a stone, in aliquots every 3 min throughout a 30‐min experiment. The scope was free to move within the vial and was targeted at visible stones. If the working channel became blocked (i.e. there was zero flow through it with suction or irrigation), and was not cleared through switching between irrigation and suction, the experiment was discontinued. No syringe flush/aspiration or mechanical manoeuvres were used to attempt to unblock the ureteroscope. At the end of the experiment, stones remaining in the vial were sieved, filtered and weighed to quantify the weight of each stone size category remaining in the vial.

As outlined above, if the stone mass had been completely cleared, the experiment was repeated with a new stone mix with the next largest stone size. For example, if a stone mix up to 125 μm (i.e. <63 and 63–125 μm) had been cleared, then the experiment was repeated with a stone mix up to 250 μm (i.e. <63, 63–125 and 125–250 μm). Between experiments, ureteroscope working channels were mechanically unobstructed with a PTFE guidewire and thoroughly washed through with 1 L saline at 500 mmHg suction pressure. After cleaning, the working channel was calibrated by measuring flow rates through it at 100‐mmHg pressure. The ureteroscopes were only reused for further experiments if flow rates through them were consistent with pre‐experiment flow rates at this set pressure – suggesting no significant debris in the working channel.

In the second experiment, we investigated the maximum stone diameter that could be suctioned by DISS through an empty 5.1‐Ch working channel vs through an 11/13‐Ch FANS ureteric access sheath. DISS suction experiments were performed using the same technique as described above, but this time without a laser fibre within the working channel. In the FANS experiments, a 11/13‐Ch 40‐cm FANS access sheath (ClearPetra; Wellead Medical, Guangzhou, China) was used in the same experimental set‐up: within a glass vial at an incline of 30**°** (Fig. 1). A 9.5‐Ch ureteroscope (Lithovue; Boston Scientific, Marlborough, MA, USA) was placed within the FANS access sheath, with the end 2 mm beyond the end of the access sheath. To replicate clinical practice, the ureteroscope was maintained in its position as a baseline, whilst the vial was filled to 40 mL and emptied (with intermittent 200‐mmHg suction) alongside the indwelling ureteroscope [14]. The ureteroscope was intermittently withdrawn beyond the suction port once every 5 min to allow an empty FANS channel. As before, the stone mixes were introduced in aliquots every 3 min throughout a 30‐min experiment. The FANS access sheath and ureteroscope were free to move inside the vial and were targeted at visible stones. The experiment was performed three times for each stone mix and the SFR was calculated as described above.

Experiments were repeated three times for each experimental group. Student's *t*‐test was used for statistics. The percentage of overall stone mass cleared was used for statistical analysis. The probability of a type I error was set at 0.05 and all testing was two‐sided.

## Results

**Fig. 2:**
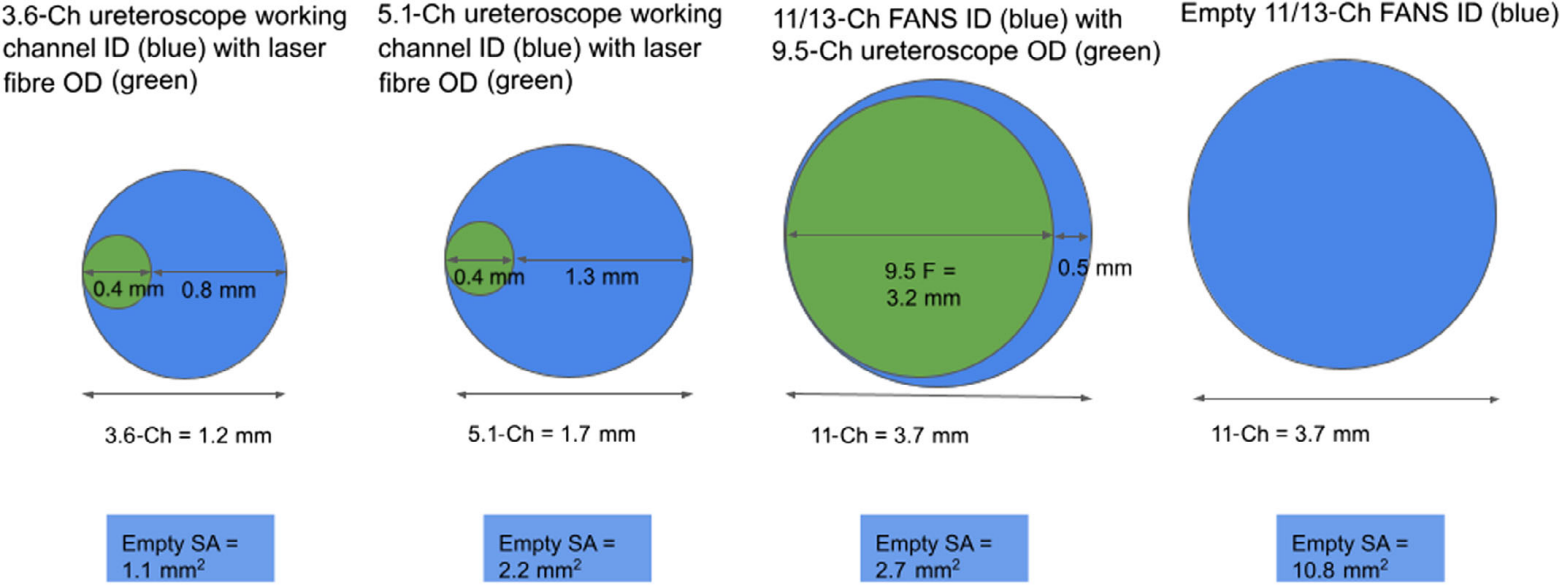
Comparison of the cross‐sectional area available for outflow and egress of stone fragments through the internal diameters (ID) of 3.6‐Ch and 5.1‐Ch working channels and suction access sheath IDs (blue circles) with indwelling laser fibres and ureteroscope outer diameters (OD; green circles). The cross‐sectional area (SA) available for egress of stones is shown in the blue boxes. The greater the cross‐sectional area available (blue areas) the lower the outflow resistance and the more space for stone fragments to pass out. FANS, flexible and navigable suction .

The maximum diameters and cross‐sectional surface areas of different ureteroscope working channel sizes and FANS access sheaths are shown in Fig. 2. With an indwelling laser fibre, a 5.1‐Ch working channel had a cross‐sectional surface area (for egress of stones) of 2.2 mm^2^, double that of a 3.6‐Ch working channel (1.1 mm^2^). An empty 5.1‐Ch working channel had a maximum internal diameter of 1.7 mm and a cross‐sectional surface area of 2.3 mm^2^. An empty 11/13‐Ch FANS access sheath had a maximum internal diameter of 3.7 mm and cross‐sectional surface area of 10.8 mm^2^.

### First Experiment

**Fig. 3:**
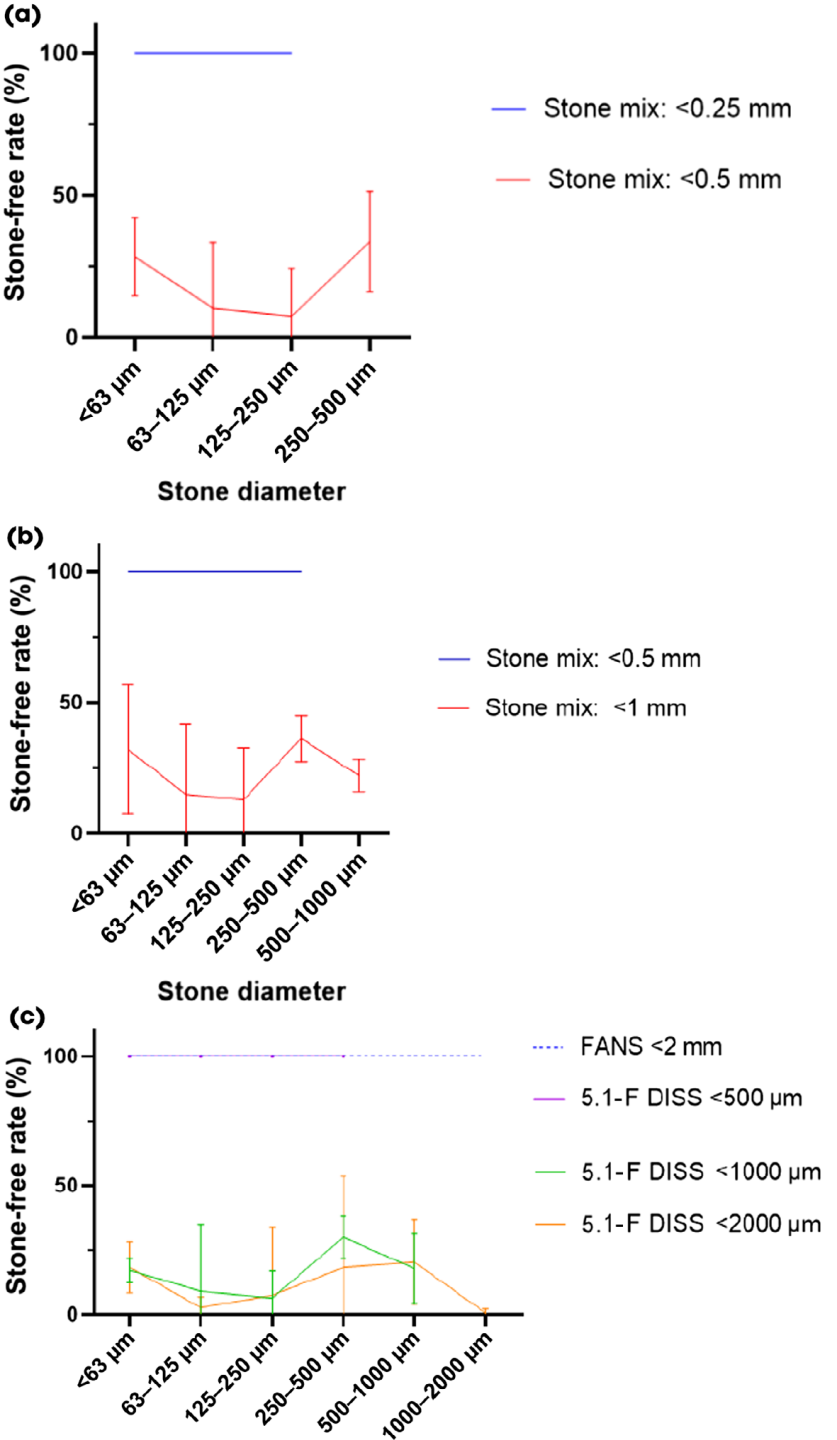
(**a**) Maximum stone fragment size that can be cleared with suction through a ureteroscope with a 3.6‐Ch working channel with an indwelling laser fibre with in‐scope suction. Stone mixes of up to 250 μm, i.e. particles <63 μm (0.55 g), 63–125 μm (0.15 g) and 125–250 μm (0.05 g), vs stone mixes of up to 500 μm, i.e. particles <63 μm (0.55 g), 63–125 μm (0.15 g), 125–250 μm (0.05 g) and 250–500 μm (0.05 g). Error bars correspond to 95% CI. (**b**) Maximum stone fragment size that can be cleared with suction through a ureteroscope with a 5.1‐Ch working channel with an indwelling laser fibre with in‐scope suction. Stone mixes of up to 500 μm, i.e. particles <63 μm (0.55 g), 63–125 μm (0.15 g), 125–250 μm (0.05 g) and 250–500 μm (0.05 g), vs stone mixes of up to 1000 μm, i.e. particles <63 μm (0.55 g), 63–125 μm (0.15 g), 125–250 μm (0.05 g), 250–500 μm (0.05 g) and 500–1000 μm (0.1 g). Error bars correspond to 95% CI. (**c**) Clearance of different stone size mixes through an empty 5.1‐Ch working‐channel in‐scope suction ureteroscope compared to an 11/13‐Ch flexible and navigable suction access sheath. Stone mixes of up to 500 μm, i.e. particles <63 μm (0.55 g), 63–125 μm (0.15 g), 125–250 μm (0.05 g) and 250–500 μm (0.05 g), stone mixes of up to 1000 μm, i.e. particles <63 μm (0.55 g), 63–125 μm (0.15 g), 125–250 μm (0.05 g), 250–500 μm (0.05 g) and 500–1000 μm (0.1 g), and stone mixes of up to 2000 μm, i.e. particles <63 μm (0.55 g), 63–125 μm (0.15 g), 125–250 μm (0.05 g), 250–500 μm (0.05 g), 500–1000 μm (0.1 g), and 1000–2000 μm (0.1 g). Error bars correspond to 95% CI.

Use of DISS through a 3.6‐Ch working channel with a laser fibre in the working channel achieved complete stone clearance of stone mixtures with particles up to 250 μm (i.e. particles of <63 μm [0.55 g], 63–125 μm [0.15 g] and 125–250 μm [0.05 g]; Fig. 3a). With the addition of stones between 250 and 500 μm (i.e. <63 μm [0.55 g], 63–125 μm [0.15 g], 125–250 μm [0.05 g] and 250–500 μm [0.05 g]), the ureteroscope repeatedly became blocked, with irrigation through the ureteroscope temporarily and intermittently unblocking the working channel (flushing the particles back into the vial). By (on average) 10 min (after the fourth aliquot) the working channel was completely blocked, preventing any flow or suction through it. Overall, 24% of the starting stone mass with particles up to 500 μm was cleared vs 100% of particles up to 250 μm (*P* = 0.01).

Use of DISS through a 5.1‐Ch working channel with a laser fibre in the working channel achieved complete clearance of stone mixtures up to 500 μm (i.e. particles <63, 63–125, 125–250 and 250–500 μm; Fig. 3b). With the addition of stones between 500 and 1000 μm (i.e. particles <63, 63–125, 125–250, 250–500 and 500–1000 μm), the ureteroscope repeatedly became blocked, with irrigation through the ureteroscope temporarily and intermittently unblocking the working channel (flushing the particles back into the vial). By (on average) 11 min (after the fourth aliquot) the working channel was completely blocked, preventing any flow or suction through it. Overall, 27% of the starting stone mass with particles up to 1000 μm was cleared vs 100% of particles up to 500 μm (*P* < 0.001).

### Second Experiment

Use of DISS through an empty 5.1‐Ch working channel achieved complete clearance of stone mixtures up to 500 μm (i.e. particles <63 μm [0.55 g], 63–125 μm [0.15 g], 125–250 μm [0.05 g] and 250–500 μm [0.05 g]), the same as with a laser fibre *in situ* (Fig. 3c). With the addition of stones between 500 and 1000 μm (i.e. particles <63 μm [0.55 g], 63–125 μm [0.15 g], 125–250 μm [0.05 g], 250–500 μm [0.05 g], 500–1000 μm [0.1 g]), the ureteroscope repeatedly became blocked, with irrigation through the ureteroscope temporarily and intermittently unblocking the working channel (flushing the particles back into the vial). By (on average) 9 min (after the third aliquot) the working channel was completely blocked, preventing any flow or suction through it. Overall, 16% of the starting stone mass with particles up to 1000 μm was cleared vs 100% of particles up to 500 μm (*P* < 0.01).

Similarly, DISS through an empty 5.1‐Ch working channel for stones up to 2000 μm (i.e. particles <63 [0.55 g], 63–125 μm [0.15 g], 125–250 μm [0.05 g], 250–500 μm [0.05 g], 500–1000 μm [0.1 g], and 1000–2000 μm [0.1 g]) resulted in the ureteroscope repeatedly becoming blocked, with irrigation through the ureteroscope temporarily and intermittently unblocking the working channel (flushing the particles back into the vial). By (on average) 6 min (after the third aliquot) the working channel was completely blocked, preventing any flow or suction through it. Overall, 14% of the starting stone mass was cleared (Fig. 3c).

By contrast, FANS aspiration through an 11/13‐Ch FANS access sheath resulted in complete stone clearance of stones up to 2000 μm (i.e. particles <63, 63–125, 125–250, 250–500, 500–1000 and 1000–2000 μm) without blockage. Overall, 100% of the starting stone mass of particles up to 2000 μm was cleared with FANS vs 14% clearance through an empty 5.1‐Ch working channel DISS ureteroscope (*P* < 0.01).

## Discussion

The results of our benchtop experiments demonstrate that, while DISS successfully clears smaller stone particles (up to 250 and 500 μm through 3.6‐Ch and 5.1‐Ch working channels), FANS demonstrates superior efficacy in clearing larger fragments (up to 2000 μm). We hypothesise that the larger working channel of the FANS access sheath allows the passage of larger fragments, while the smaller working channels of the ureteroscopes used in DISS, even without a laser fibre, limit the particle size that can be cleared.

Our results showed that an empty 5.1‐Ch working channel, despite having a maximum diameter of 1.7 mm, only achieved complete stone clearance of stones up to 500 μm. Thus, we hypothesise that it is important for the surgeon to laser the stone into suitably small particles to allow aspiration and to activate aspiration away from large particles to prevent blockage. Real‐world data suggest that, with careful laser lithotripsy, SFRs of up to 84% can be achieved with DISS [15].

In our experiments, the DISS ureteroscopes became blocked (i.e. there was no flow with either irrigation or suction pressure, with the device becoming unusable) due to particles small enough to enter the working channel but too large to pass through. For example, a 3.6‐Ch working channel with a laser had an available diameter of 0.8 mm but was blocked by 250–500‐μm particles. Similarly, a 5.1‐Ch working channel with and without a laser, with an available diameter of 1.3 mm or 1.7 mm, was blocked by 500–1000‐μm particles. Thus, whilst fragments >2 mm are too large to enter the working channel and therefore will not be cleared, particles 250–500 μm and 500–1000 μm have the potential to enter the working channel and block the ureteroscope.

Our findings suggest that DISS, both through a 3.6‐Ch and a 5.1‐Ch working channel, may be effective at improving intra‐operative vision through ‘dust’ clearance. This is in keeping with clinical studies which report good visibility and suction with use of Pusen's DISS™ ureteroscope [15, 16, 17]. Approximately 70% of stone mass created by stone laser lithotripsy is ‘dust’, defined as particles <250 μm in diameter [12, 18]. Dust floats in the irrigation fluid (sedimenting slowly), causing a ‘dust storm’ effect [18], whereas larger particles rapidly drop to the bottom of the calyx out of view [18]. Thus, it is dust which most affects visibility. Dust was cleared reliably, without blocking, through a 3.6‐Ch and 5.1‐Ch working channel with a laser fibre *in situ*.

There are several manuscripts examining the particle sizes that can be cleared with use of DISS. Schneider et al. [19] assessed the feasibility of suctioning submillimetre fragments with a standard 10‐mL Luer Lock syringe, which could generate pressures of up to 435 mmHg through an empty 3.6‐Ch working channel flexible ureteroscope. They created two ‘phantom stone’ mixes of <500‐μm vs 500–1000‐μm ‘Bego’ stones. Each stone group was then mixed in a 400‐mL beaker filled with 75 mL normal saline. The authors found that 86% of the particles in both the 500–1000‐μm and <500‐μm groups had been suctioned from the beaker, but that in the 500‐1000‐μm group the working channel became blocked four times (out of five aspirations), compared to none for the <500‐μm group. Thus, they concluded that DISS of stones ≥500 μm is not feasible via a Luer Lock syringe through a standard working channel of 3.6 Ch. In our view, there were two limitations to their study. Firstly, the smallest sieve size used was 500 μm therefore the true range of stone sizes in the <500‐μm group is not known. It is theoretically possible that all, or a high proportion, of stones were all <250 μm. Secondly, the experiment started with the entire mass of stones in the beaker (as opposed to gradual introduction as would happen throughout laser lithotripsy). This high density of Bego stone particles risks them clumping together and blocking the working channel.

Madden et al. and Chan et al. evaluated, via benchtop experiments, the maximum particle size that can be cleared through 3.6‐Ch and 5.1‐Ch DISS working channels, respectively [9, 10]. Both manuscripts concluded that particles up to 250 μm could be cleared. Our methods differ from both of these studies in two distinct ways. Firstly, in these two studies, the entire mass of Bego stone particles was introduced into the vial at the start of the experiments, whereas we introduced particles gradually in aliquots. We believe gradual introduction of particles better represents *in vivo* conditions where the particles are gradually produced by laser lithotripsy. Introducing all material at once risks artificially high particle concentrations, promoting clumping and channel blockage. Secondly, when testing the maximum particle size that could be cleared, their experiments used a homogenous particle size (i.e. 125–250‐μm Bego stones) as opposed to the full range range of particle sizes up to the maximum size in question (i.e. <63, 63–125, 125–250 μm). Laser lithotripsy produces a wide distribution of particle sizes and excluding this heterogeneity may introduce bias and limit generalisability to clinical practice [12]. Lastly, Tsaturyan et al. [20] have performed benchtop experiments demonstrating the potential to use DISS ureteroscopes to relocate 2‐ and 4‐mm fragments within the pelvicalyceal system, demonstrating further utility of the technology.

Our findings suggest that intermittent suction through a FANS access sheath has the potential to achieve complete stone clearance of fragments up to 2000 μm. These findings are in concordance with the published experimental and clinical studies on FANS SFRs. Experimentally, Chen et al. [21], in an *ex vivo* model, demonstrated marginally increased SFRs (98.5% vs 95.9%; *P* = 0.017) when using FANS vs basketing through a standard access sheath.

There have been multiple published clinical studies demonstrating high SFRs achieved with FANS [17, 22]. A meta‐analysis demonstrated higher SFRs when using suction during retrograde intra‐renal surgery [22]. A randomised controlled trial of 320 patients with renal stones <30 mm showed a higher SFR when using a FANS vs a traditional access sheath (81% vs 49%) on the first postoperative day [23]. Several prospective studies have shown high SFRs (including residual fragments <2 mm) of 96.5%–100% when using FANS [24, 25, 26]. Multiple retrospective studies have shown a similarly high SFR when using FANS compared with a traditional ureteric access sheath or no sheath [20,23, 27, 28, 29, 30, 31, 32].

Our study has a number of limitations. Firstly, our benchtop experiments may not fully replicate the complexities of *in vivo* clinical scenarios. In the clinical setting, complex anatomy and poor visibility are likely to affect the ability to find and access fragments for suction. Thus, whilst we observed complete clearance of stones up to 2000 μm with FANS and complete clearance of stones up to 500 μm with DISS, these results should be interpreted as a representation of each technique's capabilities in an ideal clinical scenario. Secondly, the use of quartz stones as a surrogate for renal stones, while an established model, may not perfectly mimic the behaviour of all stone types. Lastly, due the limited number of available ureteroscopes, we had to reuse these in different experiments. Whilst methods were put in place to minimise the bias this might introduce, it should be noted as a limitation.

Further clinical studies are needed to validate our findings and establish the optimal use of these techniques in real‐world settings.

In conclusion, we evaluated two innovative ureteroscope suction techniques, DISS and FANS, for their effectiveness in clearing renal stone particles of varying sizes. Our findings suggest that DISS through a 3.6‐Ch working channel, with an indwelling laser fibre, can clear particles up to 250 μm, while DISS through a 5.1‐Ch working channel, with or without a laser, can clear particles up to 500 μm. FANS has the potential to clear particles up to 2000 μm.

During laser lithotripsy, DISS can aspirate ‘dust’ (particles <250 μm), which is known to most affect vision [18]. However, particles >250 and >500 μm (with 3.6‐Ch and 5.1‐Ch working channels) may block the ureteroscope. FANS, through successful clearance of larger fragments, may be particularly advantageous in cases where large stone fragments are anticipated or encountered.

## Disclosure of Interests

R.M.W. and B.T. are recipients of BSC research grant funding. T.R. has no declarations. Bench test results may not necessarily be indicative of clinical performance.
